# Succinate at the Crossroad of Metabolism and Angiogenesis: Roles of SDH, HIF1α and SUCNR1

**DOI:** 10.3390/biomedicines10123089

**Published:** 2022-12-01

**Authors:** Reham Atallah, Andrea Olschewski, Akos Heinemann

**Affiliations:** 1 Otto-Loewi Research Center for Vascular Biology, Immunology and Inflammation, Division of Pharmacology, Medical University of Graz, 8010 Graz, Austria; 2 Ludwig Boltzmann Institute for Lung Vascular Research, 8010 Graz, Austria; 3 Department of Anaesthesiology and Intensive Care Medicine, Medical University of Graz, 8036 Graz, Austria

**Keywords:** succinate, succinate dehydrogenase (SDH), succinate receptor-1 (SUCNR1), hypoxia-inducible factor 1-alpha (HIF1α), angiogenesis

## Abstract

Angiogenesis is an essential process by which new blood vessels develop from existing ones. While adequate angiogenesis is a physiological process during, for example, tissue repair, insufficient and excessive angiogenesis stands on the pathological side. Fine balance between pro- and anti-angiogenic factors in the tissue environment regulates angiogenesis. Identification of these factors and how they function is a pressing topic to develop angiogenesis-targeted therapeutics. During the last decade, exciting data highlighted non-metabolic functions of intermediates of the mitochondrial Krebs cycle including succinate. Among these functions is the contribution of succinate to angiogenesis in various contexts and through different mechanisms. As the concept of targeting metabolism to treat a wide range of diseases is rising, in this review we summarize the mechanisms by which succinate regulates angiogenesis in normal and pathological settings. Gaining a comprehensive insight into how this metabolite functions as an angiogenic signal will provide a useful approach to understand diseases with aberrant or excessive angiogenic background, and may provide strategies to tackle them.

## 1. Introduction

Sprouting of new blood vessels from pre-existing ones is termed angiogenesis [1]. The importance of this process extends from fetal development to reproduction and wound repair [2]. However, in pathologies such as cancer, rheumatoid arthritis and diabetic retinopathy angiogenesis is rather a villain [1]. Angiogenesis is a complex process including vessel destabilization, endothelial cell proliferation, migration and differentiation, and eventually maturation of newly formed vessels [3]. The process is tightly regulated, and imbalance between pro- and anti-angiogenic factors can result in excessive or inadequate angiogenesis [1]. Hence, deciphering the cellular and molecular mechanisms of angiogenesis is mandatory for the development of targeted therapeutics.

Angiogenesis is an energy-requiring process. Even though endothelial cells prefer glycolysis for their energy needs, recent findings suggest that endothelial mitochondrial function is vital for vasodilatory and oxidative stress responses [4,5,6]. Perinuclear clustering of mitochondria in pulmonary arterial endothelial cells during hypoxia results in accumulation of reactive oxygen species (ROS) and subsequent vascular endothelial growth factor (VEGF) expression [7]. There is also evidence that proliferating endothelial cells increasingly depend on mitochondrial oxidative phosphorylation for their energy supply and, under growth, endothelial cells consume three times more oxygen than quiescent cells [8]. Recently, it has been demonstrated that both glycolysis and oxidative phosphorylation are crucial for purinergic receptor-mediated angiogenic responses in vasa vasorum endothelial cells [9].

Over the last few years, an enormous expansion in the knowledge and understanding of metabolic reprogramming has occurred as metabolic shifts accompanying pathologies are not just explained in the context of energy production and biosynthesis. An important role of metabolites in regulating cell behavior and fate has become a key focus of research. One major mitochondrial metabolite, succinate, has long been identified as an essential intermediate in the Krebs cycle, where succinate oxidation to fumarate occurs via the enzyme complex succinate dehydrogenase (SDH), providing electrons to the respiratory chain. SDH consists of four protein subunits encoded by SDHA, SDHB, SDHC and SDHD, all of which are encoded by nuclear genome. The enzyme complex is present in the inner mitochondrial membrane and has a matrix-facing domain, where the enzymatic activity of the complex takes place, and a hydrophobic membrane-anchoring domain [10,11]. Recently, numerous studies have provided strong evidence supporting roles of succinate outside metabolism, particularly in signal transduction and pseudohypoxia, and have been reviewed elsewhere [12].

Lately, the challenges that have imposed antiangiogenic, growth-factor-directed therapies have raised the need for a novel approach. Hence, targeting metabolism might be vital to arrest vessel growth in diseases with altered angiogenesis, specifically since the concept that endothelial cells adapt their metabolism in response to angiogenic signals has been established [13], and metabolic maladaptation of endothelial cells in diseases with endothelial dysfunction is now evident [14,15]. In this review, we aim to highlight the key mechanisms by which the metabolite succinate regulates angiogenesis as potential targets to manipulate vessel growth in these pathologies. We discuss the fundamental principles and shed light on understudied areas that require further investigation. Revealing how metabolites drive endothelial cell behavior is not only an exciting research topic but is also a therapeutically relevant one.

## 2. Angiogenesis

Angiogenesis is a complex biological process. It occurs not only under physiological conditions, but also in numerous diseases such as cancer and chronic inflammatory diseases, e.g., rheumatoid arthritis [1]. Under normal conditions, angiogenesis is a tightly regulated process that creates a network of vessels which remodel into arteries and veins [16]. While vessels are quiescent in an adult, endothelial cells lining the vessels retain a high ability to sense and respond to angiogenic stimuli in the environment [17]. Indeed, endothelial cells, in response to angiogenic signals, become motile and tip cells protrude filopodia and lead following endothelial cells [18]. Stalk cells subsequently develop fewer filopodia and retain a proliferative phenotype supporting sprout elongation. As vessel loops develop, the establishment of a basement membrane and the recruitment of mural cells stabilize newly formed vessels [17]. In addition to sprouting, angiogenesis can occur through splitting of pre-existing vessels via intussusception. In this process, transluminal pillar formation and subsequent vascular splitting occur [19]. Furthermore, induction of blood vessel growth by blood-circulating precursor cells has been proposed [20].

**Figure 1:**
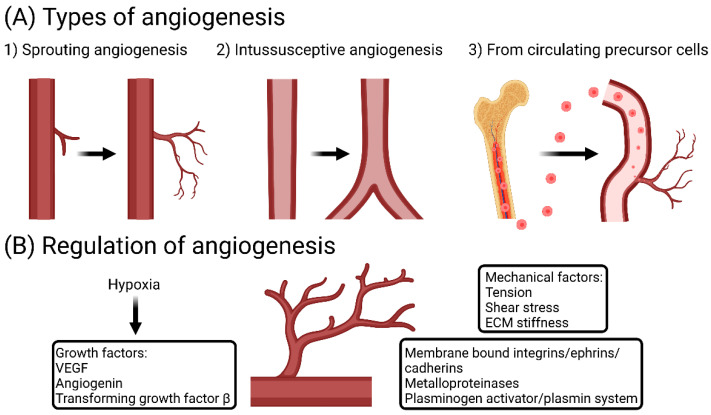
Types of angiogenesis and key regulatory mechanisms. (**A**) Types of angiogenesis. (**B**) Key factors regulating angiogenesis. VEGF: vascular endothelial growth factor, ECM: extracellular matrix.

Several biochemical and biophysical cues in the environment regulate angiogenesis. For instance, a plethora of growth factors including VEGF, angiogenin and transforming growth factor-β act as signals inducing angiogenesis [21]. In cancer, VEGF can be released by cancer cells, driven by hypoxia, and induces tumor angiogenesis by engaging VEGF receptor 2 on endothelial cells [22]. Importantly, tumor-associated macrophages (TAMs) act as a crucial source of angiogenic factors such as VEGF [23]. In addition to soluble mediators, members of membrane-bound integrins, ephrins and cadherins affect blood vessel formation together with metalloproteinases and the plasminogen activator/plasmin system [1]. In parallel, mechanical cues such as extracellular matrix stiffness, shear stress and tension regulate angiogenesis. However, they are less studied and require more investigation [24]. Figure 1 summarizes types of angiogenesis and key regulatory factors.

## 3. Metabolic Regulation of Angiogenesis

In diseases such as cancer, processes like metabolism and angiogenesis are critical for tumor progression. Increased resistance of certain tumors to antiangiogenic therapies has been linked to metabolic symbiosis in cancer cells denoting that metabolic adaptations have a significant impact on cellular response to antiangiogenic therapies [25]. Additionally, recent data demonstrated that growth factors induce metabolic changes in endothelial cells that drive their phenotype during angiogenesis [26]. Indeed, distinct metabolic signatures have been noted among tip cells, stalk cells and quiescent endothelial cells during angiogenesis [27]. For instance, administration of etomoxir, a drug targeting carnitine palmitoyl transferase in mitochondria, to juvenile mice with retinopathy of prematurity ameliorated retinal neovascularization [28]. These studies highlight the important link between metabolic rewiring and angiogenesis. In the next sections, we will focus on a mitochondrial metabolite, succinate, and provide an overview of the different mechanisms by which accumulation of this metabolite might regulate angiogenesis. Adding the contribution of metabolites such as succinate as an extra dimension to our understanding of angiogenesis will provide new opportunities to develop therapeutics that target this critical process.

## 4. Succinate in Biological Fluids and Tissues between Health and Disease

Succinate is generated in the mitochondria, but it is exported to the cytosol by dicarboxylate carrier localized in the inner mitochondrial membrane [29], and voltage-dependent anion channel (VDAC/porin) in the mitochondrial outer membrane [30]. Succinate can be measured in blood at concentrations of 2–20 µM [31,32]. However, succinate concentration can rise up to mM range under pathological conditions such as ischemia [33]. Detailed tracing of the mechanisms of succinate accumulation and the patho/physiological consequences of its accumulation is beyond the focus of this review. Accordingly, in this section we will only highlight selected examples.

In the serum of patients with myocardial infarction plus coronary artery disease, elevated succinate concentrations in the mM range were found in contrast to undetectable levels in age-matched controls [33]. Likewise, circulating levels of succinate were higher in patients with head and neck squamous cell carcinoma [34], and in Cowden Syndrome patients with germline mutations of phosphatase and tensin homolog (PTEN), SDHB or SDHD [35], than in the healthy controls. A recent publication highlighted a positive association between plasma succinate levels, visceral adipose tissue mass, triglycerides and pro-inflammatory omega-6 oxylipins levels. Hence, succinate levels in plasma might reflect cardiovascular status in young adults [36]. Furthermore, Zhu et al. unraveled a panel of five serum metabolites, including succinate, which could be used for monitoring colorectal cancer progression by sequential metabolite ratio analysis of serial serum samples of patients [37]. Likewise, Gong et al. identified a panel of biomarkers, including significantly elevated serum level of succinate, which could distinguish hepatocellular carcinoma from healthy controls and patients with HBV-cirrhosis [38].

Succinate accumulation was also reported in brown adipose tissue upon exposure to cold and activated thermogenic respiration in brown adipocytes [39]. Furthermore, serum and intestinal levels of succinate were elevated in patients with Crohn’s disease, demonstrating a role of this metabolite in intestinal inflammation [40]. In hemorrhagic shock, succinate accumulated in plasma and lung tissue driven mainly by glutaminolysis [41]. Furthermore, we recently demonstrated increased succinate concentrations in gestational diabetic term placentas relative to matched controls, denoting that succinate accumulation is a hallmark of this pregnancy pathology [42]. Similarly, increased succinate levels in prostatic cancer tissue accompanied with reduced SDHD expression were described as an important metabolic feature in a cohort of prostate cancer patients. Notably, this study applied a strategy to avoid systematic bias due to tissue heterogeneity by matching the average stroma tissue content in all samples [43]. A further study using benign and prostate cancer cells demonstrated that loss of PTEN was associated with increased succinate levels and enhanced succinate-driven mitochondrial respiration [44]. Collectively, these studies support the idea that monitoring succinate levels in the circulation or in the tissue could provide a new tool for diagnosis or therapeutic intervention of pathologies accompanied with metabolic aberrations.

In addition to plasma and tissues, succinate can also be found in other biological fluids such as urine. Succinate was among 16 metabolites that were distinctly detected in urine samples of patients with gastric cancer [45], colorectal cancer [46] and esophageal cancer [47,48]. In bladder cancer, mass-spectrometry-based metabolomics of urine samples suggested succinate and other compounds such as palmitoyl-sphingomyelin, lactate and adenosine as the most putative markers differentiating cancer from non-cancer samples [49]. Cala et al. used metabolomics and lipid fingerprinting to investigate urinary metabolite alterations of breast cancer in Hispanic women. Overall reduction in the expression of Krebs cycle metabolites was observed and a combination of succinate and dimethyl-heptanoyl-carnitine was found as a potential urinary biomarker for breast cancer [50].

Additionally, in human subjects with proliferative diabetic retinopathy, vitreous succinate and VEGF were increased. In this study, the authors suggested a positive feedback mechanism between VEGF and succinate since VEGF inhibition decreased succinate [51]. Moreover, in early-stage oral squamous cell carcinoma, succinic acid content, among various metabolites, in saliva was increased compared to healthy people [52]. Higher fecal succinate levels were measured in colorectal cancer patients compared to the healthy controls and this altered metabolic profile was proposed to distinguish colorectal cancer even at early stages [53].

Similar findings were described in rodent models of hypertension and metabolic disease, where circulating succinate concentration was increased [31]. Elevated succinate levels were also described in diabetic mice kidney and urine [54], as well as diabetic rat retinas [55]. In kidneys of diabetic mice, succinate acted as a suppressor for mitochondrial β-oxidation via elevation of the mitochondrial NADH/NAD^+^ ratio. Hence, accumulation of succinate caused lipid accumulation in diabetic kidneys [56]. In the rat liver, non-enzymatic formation of succinate via α-ketoglutarate decarboxylation in mitochondria under oxidative stress was unraveled and resulted in decreased ROS levels [57], while increased levels of succinate due to inverse catalysis of SDH and increased oxidative stress and neuronal damage were demonstrated in a rat model of status epilepticus induced by kainic acid [58].

At the cellular level, succinate accumulation has been reported in immune cells subjected to inflammatory stimuli. For instance, in LPS-stimulated macrophages, succinate concentration was increased and glutamine-dependent anaplerosis was the main source of succinate in addition to GABA shunt [12,59]. Similarly, elevated succinate occurred in synovial macrophages and fibroblasts subjected to LPS stimulation and low oxygen, respectively. These findings corroborated a role of succinate in modulating the inflammatory response in rheumatoid arthritis [60].

Another critical source of succinate in the body is the microbiome, particularly gut microbiota, which serves as a great contributor of succinate in the body [61]. In the mammalian gut, bacteria belonging to the Bacteroidetes phylum produce major amounts of succinate as a byproduct of anaerobic fermentation [62]. Indeed, the succinate pathway is the major route for the formation of propionate from dietary carbohydrates, and is found mainly in *Bacteroides* spp. and *Prevotella* spp. [63]. Interestingly, both spp. were prevalent in fecal samples of type 2 diabetes patients [64]. Hence, it is arguable that altered microbiota could be a potential source of increased levels of succinate in obese and diabetic individuals [65]. Furthermore, Rosenberg et al., using metabolic profiling and dual RNA sequencing, demonstrated that succinate accumulation in macrophages was sensed by intracellular *Salmonella Typhimurium* to promote its virulence [66]. Likewise, *Mycoplasma arginini* infection of VM-M3 cancer cells enhanced the Warburg effect and succinate production in mitochondria, and release into the extracellular milieu in a mechanism that was independent of the cytosolic glucose-driven lactate production [67].

**Figure 2:**
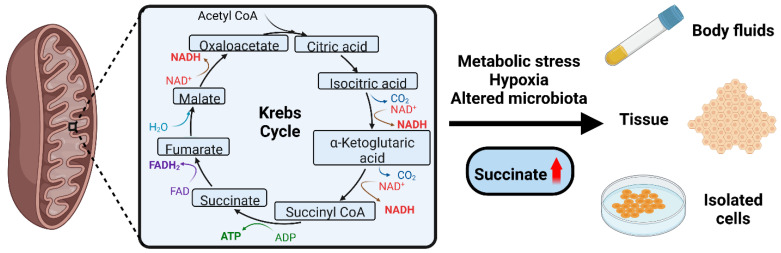
Succinate accumulation occurs in metabolic stress conditions. Succinate is an intermediate metabolite in the Krebs cycle. In conditions of metabolic stress/hypoxia/microbiome alterations, succinate concentrations rise beyond physiological values. Succinate can be measured in biological fluids or tissues, or in isolated cells exposed to stress. Red arrow denotes increased succinate levels.

In summary, under physiologically normal circumstances no accumulation of succinate occurs. However, in metabolic stress conditions and/or hypoxia or altered microbiome composition succinate concentrations rise sharply. This increase could be measured in biological specimens like plasma as well as tissues or using isolated cells challenged with pro-inflammatory stimuli. This was evident using human material and in animal studies. Figure 2 demonstrates the Krebs cycle where succinate is produced and the key concept of succinate elevation under stress conditions.

## 5. SDH Alterations as a Cause of Succinate Accumulation

Mitochondrial SDH oxidizes succinate to fumarate and provides electrons to the electron transport chain. However, in many pathologies alterations in SDH occur leading to increased succinate concentrations. For instance, in different cancer types such as paraganglioma/pheochromocytoma (PGL/PCC), renal carcinoma, ovarian cancer, neuroblastoma and gastrointestinal stromal tumor, succinate accumulation in the local environment occurs due to mutations in SDH [68]. Mutations of the B and D subunits of SDH are commonly reported, whereas mutations of the A and C subunits occur to a lesser extent [69,70]. SDH mutations lead to enzymatic inhibition of multiple α-ketoglutartate-dependent dioxygenases such as histone demethylases, prolyl hydroxylases, collagen prolyl-4-hydroxylases and the TET (ten-eleven translocation) family of 5-methylcytosine (5 mC) hydroxylases. Consequently, alterations of genome-wide histone and DNA methylation occur and contribute to tumorigenesis [71].

In addition to mutations, processes such as methylation of SDH gene promoter and expression of specific miRNAs can affect the stability and activity of SDH [72,73,74]. Increased expression of miRNA-210, -31 and -378, which specifically target SDH mRNA, was reported in cancer cells after radiotherapy [75]; miR-210 was also highly expressed at a late stage of lung cancer, and targeted SDHD resulting in significant alterations in cell metabolism and survival in addition to increased hypoxia-inducible factor1 (HIF1) activity [76]. Another study by Merlo et al. identified a signaling axis of HIF1α/miRNA-210/iron–sulfur cluster scaffold protein (ISCU) in a subset of head and neck paragangliomas that might have an impact on SDHB protein stability by a mechanism independent of SDH mutations [77]. While miRNA-31 targeted SDHA mRNA in induced pluripotent stem cells altering ROS generation, mitochondrial membrane potential and mitochondrial mass [78], miRNA-378 in breast cancer cells regulated SDHB mRNA resulting in a metabolic shift away from oxidative metabolism and cell proliferation [79]. Furthermore, non-sense mutations during RNA editing led to decreased mRNA expression of the SDH gene as an adaptation mechanism to hypoxia in monocytes [80].

Phosphorylation and acetylation post-translational modifications can also regulate the activity of SDH. In conditions of oxidative stress, SDHA phosphorylation was increased and subsequently induced complex-II-dependent respiration [81]. In contrast, dephosphorylation of SDH by PTEN-like mitochondrial phosphatase-1 (PTPMT1) limited SDH activity and was proposed to play a role in glucose homeostasis [82]. Furthermore, loss of sirtuin 3, a NAD-dependent deacetylase, hampered the enzymatic activity of SDH, implying a role of sirtuin 3 as a regulator of SDH activity [83]. Oncogenic transcription factors such as Myc induced acetylation-dependent deactivation of SDHA, which resulted in cellular succinate accumulation and consequently trimethylated histone H3 Lys 4 (H3K4me3) activation and expression of tumor-specific genes [84]. Another post-translational modification that is influenced by succinate itself is lysine succinylation, which extensively regulates metabolic enzyme activities in mitochondria [85]. Notably, SDH lysine succinylation increased its activity and hence was proposed as an auto-regulatory mechanism of succinate levels in mitochondria [86,87].

**Figure 3:**
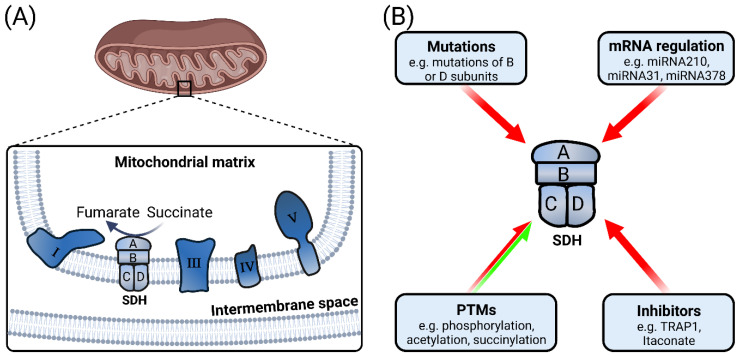
SDH is a key enzyme metabolizing succinate in the mitochondria. (**A**) SDH complex is composed of 4 subunits and is part of the electron transport chain in the inner mitochondrial membrane. (**B**) SDH expression and/or activity can be regulated by different effectors. Red arrows indicate that these regulators reduce SDH expression and/or activity, while the split red and green arrow indicates possible upregulation or downregulation of SDH activity. PTMs: post-translational modifications, TRAP1: tumor-necrosis-factor-receptor-associated protein 1.

Another mechanism of SDH regulation is through tumor-necrosis-factor-receptor-associated protein 1 (TRAP1), a mitochondrial molecular chaperone also identified as heat shock protein 75. TRAP1 can bind to and inhibit SDH and subsequently induces succinate accumulation [88]. Similarly, itaconate inhibits SDH resulting in succinate accumulation. In vivo treatment with exogenous itaconate increased succinate levels, thus inhibiting mitochondrial respiration, and exerting anti-inflammatory effects during macrophage activation [89,90]. Figure 3 illustrates the localization of SDH enzyme complex and summarizes key factors regulating its expression and/or activity.

In conditions of ischemia/hypoxia, SDH acts in reverse mode to reduce fumarate to succinate where fumarate is derived mainly from malate/aspartate shuttle and AMP-dependent activation of the purine nucleotide cycle. Upon reperfusion after ischemia, rapid oxidation of accumulated succinate by SDH prompted extensive ROS generation by reverse electron transport at mitochondrial complex I [91]. Indeed, succinate accumulation was a common feature of mouse, pig and human ischemic hearts. In this study, the authors suggested that cooling the tissue before transplant slowed succinate generation, thereby reducing tissue damage upon reperfusion caused by the production of mitochondrial ROS [92]. Interestingly, ischemic preconditioning had no effect on succinate accumulation or oxidation during murine cardiac ischemia/reperfusion injury [93]. Notably, canonical Krebs cycle activity, partly supported by aminotransferase anaplerosis and glycolysis from glycogen, was proposed as a primary mechanism for succinate accumulation in ischemic hearts in addition to the reverse action of SDH [94]. In cardiomyocytes, a lipid insult resulted in intra- and extracellular succinate accumulation, which consequently inhibited pyruvate dehydrogenase activity and aggravated ischemia/reperfusion injury [95]. Similarly, in ischemia/reperfusion-affected rat kidneys, accumulation of succinate in the cytosolic fraction occurred and was associated with increased H_2_O_2_ generation mediated by complex II [96]. In another study, treatment with melatonin prevented SDHB-induced succinate accumulation and reduced succinate-mediated growth of uterine endometrial cancer cells in vitro and in vivo. Hence, targeting SDH could ameliorate succinate-mediated cancer progression especially in patients who express abnormally reduced levels of SDHB [97]. In contrast to these observations, a study by Wijermars et al. demonstrated that no accumulation of succinate occurred during human renal graft procurement. Surprisingly, tissue succinate content progressively decreased with increasing graft ischemia time. These findings highlighted the challenge of the translation potential of data generated in rodent models of ischemia/reperfusion to humans [98].

Collectively, SDH enzyme complex is responsible for the metabolism of succinate into fumarate in the Krebs cycle. Alterations in SDH expression and/or activity due to mutations or expression of miRNAs that target SDH subunits, or post-translational modifications or the presence of enzyme inhibitors like TRAP1 result in succinate accumulation. Hence, SDH is an essential regulator of succinate concentrations.

In Section 6 and Section 7, we summarize two critical consequences of succinate accumulation, which include the induction of pseudohypoxic responses due to HIF1α stabilization and signaling via a metabolite sensor named accordingly SUCNR1. Both pathways can result in excessive angiogenesis as discussed in Section 8.

## 6. Succinate Accumulation and Induction of Pseudohypoxia

**Figure 4:**
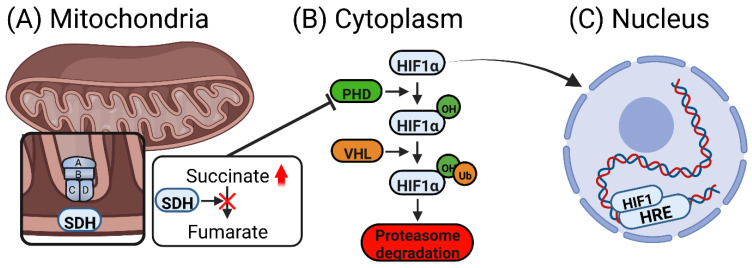
Succinate stabilizes HIF1α by inhibiting its degradation. Succinate inhibits PHD leading to HIF1α stabilization and translocation to the nucleus. Subsequently, assembly of the HIF1 complex occurs and induction of hypoxic responses takes place. Red arrow denotes increased succinate concentration. HIF: hypoxia-inducible factor, PHD: prolyl hydroxylase, VHL: von Hippel–Lindau protein, Ub: ubiquitin protein, HRE: hypoxia response element.

An important consequence of succinate accumulation in the cytosol is the inhibition of prolyl hydroxylase (PHD) [30]. This induces HIF1α activation and stabilization, and subsequent upregulation of target genes containing hypoxia response elements (HRE), including angiogenic genes like VEGF [70]. This state is referred to as pseudohypoxia since hypoxic responses are initiated under normal oxygen levels through transcription factors such as HIF1α [99]. The process of inhibition of PHD by succinate could be reversed in vitro by the addition of α-ketoglutarate [30,100]. Figure 4 demonstrates this pathway.

Another mechanism by which succinate can stabilize HIF1α is through ROS induction [12]. Indeed, pharmacological inhibition of SDH, as well as RNA interference of SDHB, increased ROS production and subsequently resulted in HIF1α stabilization [101,102]. Furthermore, Guzy et al. showed that antioxidants abolished the effects of succinate on HIF1 activation [101]. ROS stabilizes HIF1α via oxidation of Fe^2+^ (an essential PHD cofactor) to Fe^3+^ thus limiting its activity [103]. However, contradicting results demonstrated that alterations in SDHA, SDHB and SDHD genes did not increase ROS production, and attributed HIF1α accumulation and activation to succinate-mediated PHD inhibition [30,104]. Supporting these observations, Tseng et al. described reduced ROS levels in cells with *SDHB*-knockdown and elevated ROS in SDHB-overexpression cells compared to the parental human hepatocellular carcinoma cell line [105].

In endothelial cells, HIF1α regulates the transcription of numerous growth factors and hence plays a crucial role in angiogenesis. Overexpression of HIF1α in endothelial cells in vitro, under non-hypoxic conditions, induced morphological changes that were comparable to those induced by hypoxia, such as invasion and capillary-like tube formation [106]. Furthermore, conditional deletion of HIF1α in endothelial cells in vivo profoundly hindered blood vessel growth in solid tumors corroborating the crucial role of HIF1α in endothelial cell function [107].

In addition to induction of angiogenic genes, the implications of HIF1α activation have been extensively studied in immune contexts. For instance, HIF1α in LPS/IFNγ-stimulated macrophages induced a shift of the mitochondrial function from ATP production to ROS production, making the cells more vulnerable to DNA damage and necroptosis [108], while myeloid-specific HIF1α overexpression in a mouse model induced M1 polarization in macrophages via promoting glycolytic metabolism [109]. In rheumatoid arthritis macrophages, HIF1α expression was increased and was a prerequisite for IL-1β production [110]. In T cells, HIF1 enhanced TH17 development and attenuated Treg development suggesting a role of metabolic modulation, possibly via succinate, in T-cell-based diseases [111]. In mouse dendritic cells, LPS induced HIF1α gene and protein expression as well as HIF1α target genes such as VEGF. Additionally, HIF1α played a major role in interferon-α and -β production in dendritic cells and HIF1α-deficient cells showed hindered ability to activate T cells [112]. These findings extend the implications of HIF1α activation to include both innate and adaptive immune responses, and suggest that succinate is vital for both through its effect on HIF1α stabilization.

In summary, elevated succinate concentrations in cells result in stabilization of HIF1α and thus induction of genes with HRE takes place. This can occur under normal oxygen conditions. Accordingly, this phenomenon is called pseudohypoxia. In addition to angiogenesis, the implications of HIF1α stabilization include immune response and ROS production.

## 7. Succinate Signaling via SUCNR1

SUCNR1 is a member of the superfamily of G-protein coupled receptors (GPCRs). This class of receptors is currently the target of around 60% of marketed therapies [113]. Upon ligand binding to GPCR, conformational changes and recruitment of their G-protein partners occur inducing diverse biological responses that include migration, growth and cell division [114]. It was previously thought that SUCNR1 belonged to the purinergic receptors and was predicted to bind to purinergic ligands due to its high sequence homology to P2Y receptors [115]. However, SUCNR1 was linked to succinate in a milestone study that demonstrated that intravenous succinate infusion induced an increase in blood pressure, a response that was abrogated in SUCNR1-deficient animals [116].

The half-maximal response concentration of human SUCNR1 is about 56 ± 8 µM [116], proposing that only a small increase in circulating succinate levels could result in full receptor activation. Recently, Geubelle et al. identified cis-epoxysuccinic acid and cis-1,2-cyclopropanedicarboxylic acid as SUCNR1 agonists with similar efficacy to succinic acid. Surprisingly, cis-epoxysuccinic acid had 10- to 20-fold higher potency than succinic acid on SUCNR1 [117]. To date, only few SUCNR1 antagonists have been developed [118,119], among which a high-affinity SUCNR1 antagonist is denoted NF-56-EJ40 [119]. It significantly inhibited succinate/IL-1β signaling in HUVECs and macrophages [120]. Furthermore, inhibition of SUCNR1 by NF-56-EJ40 substantially reduced succinate-mediated gene expression in primary human M2 macrophages [121].

The studies investigating the signaling machinery downstream of SUCNR1 showed significant discrepancy. For instance, in human embryonic kidney (HEK293) cells, SUCNR1 coupled to both Gi and Gq proteins [116]. Similar signaling pattern was observed in polarized Madin Darby Canine Kidney (MDCK) cells where SUCNR1 used both the Gq/11 and Gi/o pathways to increase intracellular calcium and induce extracellular-signal-regulated kinase 1 and 2 (ERK1/2) phosphorylation [122]. The activation of Gi, quantified as a reduction of cAMP levels, upon succinate binding to SUCNR1 was demonstrated in both heterologous and native systems [123,124,125,126,127]. In contrast, SUCNR1 activation of the Gαs leading to increased PKA activity was described in macrophages and mediated an anti-inflammatory response [128]. Interestingly, some publications reported that [Ca^2+^]i mobilization upon SUCNR1 activation was a consequence of PLC-β activation by the βγ dimer, as opposed to classical Gαq stimulated Ca^2+^ mobilization [126,127].

**Figure 5:**
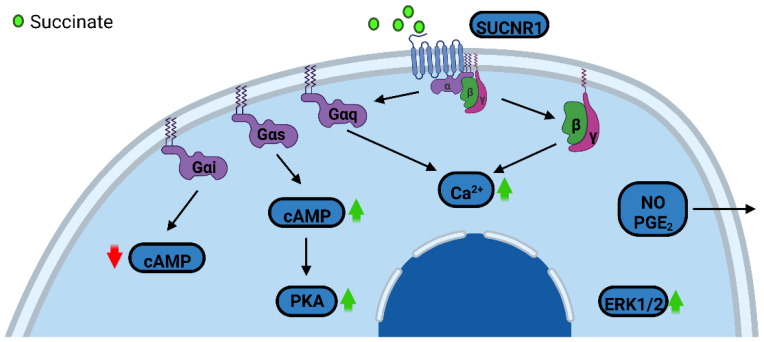
SUCNR1 downstream signaling machinery. Distinct G proteins pair with SUCNR1 mediating different cellular responses. cAMP: cyclic adenosine monophosphate, PKA: protein kinase A, ERK1/2: extracellular-signal-regulated kinases 1 and 2, NO: nitric oxide, PGE_2_: prostaglandin E_2_. Red arrow denotes reduced levels while green arrows denote increased levels or activity.

Activation of mitogen-activated pathway (MAP) kinases, especially ERK1/2, upon SUCNR1 stimulation has been described in numerous cell models such as in HEK293 cells [116,127], MDCK [122], immature dendritic cells [129], retinal ganglion neuronal cell line [130], TF-1 (human erythroleukemia) cell line [123], cardiomyocytes [131] and HUVECs [42,132]. Additionally, induction of nitric oxide production and prostaglandin E_2_ secretion upon activation of SUCNR1 were also noted [129,133]. Figure 5 illustrates the distinct signaling pathways downstream of SUCNR1.

In HEK293 cells, SUCNR1 was internalized into vesicular structures upon succinate stimulation [116]. However, in polarized MDCK cells SUCNR1 was rapidly desensitized and re-sensitized but not internalized [122]. Similarly, desensitization of SUCNR1 occurred in platelets [124]. However, interesting data demonstrated that coupling of activated SUCNR1 to arrestins 2 and 3 was very weak [127,134]. Therefore, it has been proposed that homologous desensitization and internalization of SUCNR1 occurred independent of arrestins. The distinct and even contradicting observations regarding SUCNR1 signaling and trafficking might be reflecting distinct G protein partners among different cell types or artifacts due to overexpression of the receptor. Hence, further investigation of SUCNR1 signaling and trafficking, especially in native and physiologically relevant systems, is warranted.

The pathophysiological implications of succinate–SUCNR1 signaling are evident in conditions where local stress affects cellular metabolism such as hyperglycemia, ischemia and hypoxia, and can be reviewed elsewhere [135]. Thus, it is plausible that SUCNR1 acts as a metabolic sensor, modulating cellular functions in response to succinate. Some of these inferences, in the context of angiogenesis, are addressed below.

## 8. Succinate as a Regulator of Angiogenesis

As mentioned before, accumulated succinate in the cytosol stabilizes HIF1α and subsequently induces the expression of genes with HRE, which include angiogenesis regulatory genes such as VEGF. Succinate can also be released to the extracellular space where it activates SUCNR1 and mediates a wide range of responses including angiogenesis. In this section, we highlight studies that particularly demonstrated a role of succinate in angiogenesis both in normal and pathological contexts (summarized in Table 1). We also cover studies that implicate an indirect role of succinate in angiogenesis via immune cells, particularly macrophages.

In tumor tissues with SDH mutations such as paragangliomas and phaeochromocytomas, increased succinate concentrations together with increased expression of HIF1α and associated angiogenic genes such as VEGF were reported [136,137,138]. Similarly, elevated succinate was measured in human gastric cancer tissues in comparison to paracancerous tissues, where the succinate–SUCNR1 axis induced tumor angiogenesis via regulation of ERK1/2 and STAT3 signaling [132]. As stated earlier, circulating succinate was elevated in patients with head and neck squamous cell carcinoma, along with increased expression of SUCNR1, HIF1α, SDHA and SDHB in the tumor tissue relative to matched normal mucosa, further highlighting a role of succinate as oncometabolite [34]. Another study demonstrated that targeting HIF1α was a promising approach to face chemo-resistance in colorectal cancer. In this study, the DNA-demethylating agent zebularine induced HIF1α protein degradation via hydroxylation, reduced tumor angiogenesis and potentiated the anticancer effect of oxaliplatin in an induced colorectal cancer model [139]. In melanoma cell lines, microphthalmia-associated transcription factor (MITF) activated SDHB expression and inhibited succinate accumulation and the consequent stabilization of HIF. Hence, it ameliorated the pseudohypoxic response by regulating succinate levels [140]. Indeed, a correlation between increased expression of HIF1α and HIF2α, and VEGF expression was described in nodular malignant melanomas [141]. Similarly, in triple-negative breast cancer, increased activity of the HIF1 pathway was detected [142], and collagen prolyl 4-hydroxylase 1 (P4HA1) was demonstrated to enhance HIF1α stability by modulating α-ketoglutarate and succinate levels. Therefore, targeting collagen P4H could be a novel strategy to hinder tumor progression and increase the sensitivity of triple-negative breast cancer to chemotherapeutic agents [143]. Collectively, these studies demonstrate that succinate is a critical regulator of tumor progression and angiogenesis and shed lights on SDH, HIF1α and SUCNR1 as valid targets to manipulate angiogenesis in cancer.

**Table 1:** List of studies that investigated a direct role of succinate in angiogenesis and/or tumorigenesis.

Tissue/Cells	Pathological Context	Mechanism/Effectors	References
Neuroendocrine	Pheochromocytoma paraganglioma	SDH mutation	[136,137,138]
Breast	Triple-negative breast cancer	HIF1α stabilization	[143]
Melanoma cell line	Melanoma	HIF1α stabilization	[140]
Intestine	Gastric cancer	SUCNR1-mediated ERK1/2 and STAT3 phosphorylation	[132]
	Colorectal cancer	HIF1α stabilization	[139]
Oral cavity, pharynx and larynx	Head and neck squamous cell carcinoma	Increased expression of SUCNR1, HIF1α, SDHA and SDHB	[34]
Placenta	Gestational diabetes	SUCNR1-mediated ERK1/2 phosphorylation	[42]
Retina/retinal ganglion cells	Diabetic retinopathy	SUCNR1-mediated ERK1/2/COX-2 signaling	[55]
	Proliferative ischemic retinopathy	SUCNR1 activation	[144,145]
Synovium	Rheumatoid arthritis	HIF1α induction and via SUCNR1	[60]
Peripheral limb muscles	Acute peripheral ischemia	Increased SUCNR1 expression	[146]
Brain	Hypoxia/ischemia brain injury	SUCNR1 regulation of prostaglandin E_2_–prostaglandin E receptor 4	[147]

As endothelial cells are central players in angiogenesis, we recently demonstrated that succinate, via SUCNR1, induced an angiogenic phenotype in endothelial cells in migration and sprouting assays, and by upregulating VEGF gene expression, a response which could be of pathological relevance in placental hypervascularization in gestational diabetes [42]. Furthermore, in diabetic rat retinas, knockdown of SUCNR1 hindered the activities of ERK1/2 and cyclooxygenase-2 (COX-2) and reduced the expression of PGE_2_ and VEGF [55]. Likewise, succinate regulated angiogenesis in hypoxic retinas of rodents via SUCNR1 in retinal ganglion cells, in the settings of both normal retinal development and proliferative ischemic retinopathy. Indeed, retinal ganglion cells in response to succinate upregulated the production of angiogenic factors such as VEGF [144]. A recent publication verified that SUCNR1 in retinal ganglion cells was localized at the endoplasmic reticulum and that this localization was necessary for its angiogenic regulatory role [145].

In arthritic synovium, succinate accumulation resulted in angiogenesis and exacerbated inflammation through HIF1α induction and via SUCNR1. Furthermore, targeting SDH prevented succinate accumulation and inhibited angiogenesis in rheumatoid arthritis [60]. In addition, succinate injection promoted earlier angiogenesis after acute peripheral ischemia in mice, inducing more effective revascularization with prolonged response [146]. Evidence also supported that succinate/SUCNR1 enhanced post-hypoxia/ischemia vascularization and reduced infarct size in a mouse model of newborn hypoxia/ischemia brain injury acting through prostaglandin E_2_–prostaglandin E receptor 4 to govern expression of major angiogenic factors. In this study, cyclooxygenase inhibitor and selective prostaglandin E receptor 4 antagonist both hampered succinate-induced VEGF gene expression [147].

Another indirect mechanism by which succinate regulates angiogenesis is via macrophages. In fact, TAMs are promotors of angiogenic neovascularization and this phenotype could be induced by hypoxic-tumor-cell-derived succinate and lactate [148]. Macrophages could sense succinate in the environment via SUCNR1, which then drives polarization of macrophages into TAMs. In a study by Wu et al. phosphoinositide 3-kinase (PI3K) signaling downstream of SUCNR1 participated in succinate-induced TAM polarization [149]. TAMs enhance cancer angiogenesis through the release of several pro-angiogenic factors such as VEGFA [150]. In some tumors, TAMs seemed to be a major source of MMP9, which mediate extracellular matrix degradation and release of bioactive VEGFA. Other angiogenic factors released by TAMs include basic fibroblast growth factor (bFGF), thymidine phosphorylase (TP), urokinase-type plasminogen activator (uPA) and adrenomedullin (ADM) [151].

In a tissue environment, when pro-angiogenic factors outbalance anti-angiogenic ones, an angiogenic switch in endothelial cells is turned on, resulting in the activation, proliferation and migration of these cells into tube-like structures [152,153]. Supporting these data, targeting the enzyme glutamine synthetase, which synthesizes glutamine from glutamate, in M2-polarized macrophages skewed their polarization towards M1-like phenotype and hindered their ability to foster endothelial cell branching, and thus their angiogenic potential [154]. Interestingly, TAM-derived factors such as transforming growth factor-β could inhibit SDH in the tumor cells, which resulted in an accumulation of succinate and subsequent HIF1α stabilization. TAM depletion–repletion in a 4T1 mouse model of breast cancer corroborated that TAMs promoted HIF-associated vascularization [155].

Taken together, there is substantial evidence supporting the role of succinate in regulating angiogenesis via HIF1α and SUCNR1 in different settings. Furthermore, SDH is an important target regulating succinate levels and subsequently succinate-induced angiogenesis.

## 9. Conclusions and Future Perspectives

**Figure 6:**
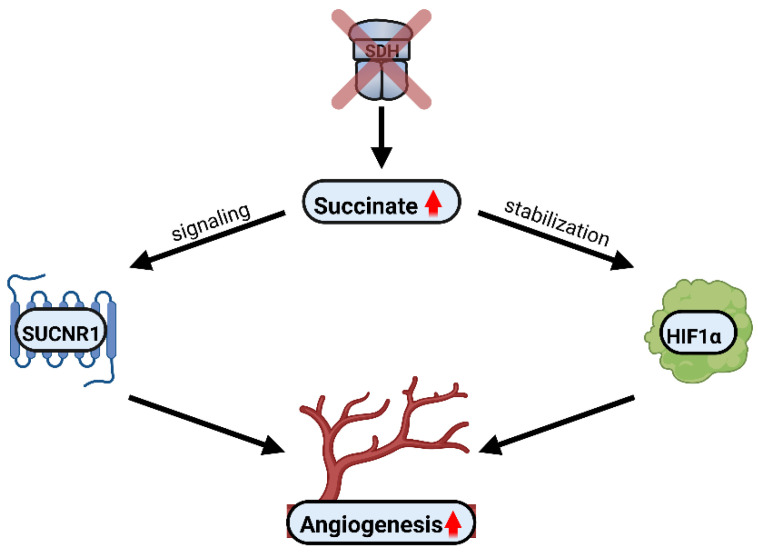
SDH, HIF1α and SUCNR1 are key targets modulating angiogenesis. In conditions where the expression and/or the activity of SDH are hindered, succinate levels increase. Accumulation of succinate can induce angiogenesis via two mechanisms, either through HIF1α stabilization or by SUCNR1 signaling. This has been shown in many pathological settings so far and is yet to be exploited to ameliorate pathological angiogenesis. Red arrows stand for elevated succinate concentrations and increased angiogenesis.

In the Krebs cycle, succinate is metabolized to fumarate by the enzyme complex SDH, resulting in energy production. In conditions where the expression and/or activity of SDH are hindered, succinate concentrations rise beyond physiological levels. This can have implications for cellular behavior including succinate-mediated regulation of angiogenesis. Succinate on the one hand can stabilize HIF1α by inhibiting its degradation and on the other hand can bind to and activate its GPCR partner, SUCNR1. Both pathways have become evident as mechanisms inducing angiogenesis in physiological and pathological settings. Hence, SDH, HIF1α and SUCNR1 are vital checkpoints in the tuning of angiogenesis, as shown in Figure 6.

Despite the remarkable progress in understanding the role of succinate as an angiogenic signal, more research is required to further unravel the interplay between different metabolites in the environment, especially in endothelial cells. Furthermore, means to restore the activity of SDH and shuttles to ameliorate succinate accumulation need to be explored. So far, it seems that, in diseases such as cancer, succinate is released by hypoxic cells and activates SUCNR1 on endothelial cells. Whether endothelial cells accumulate and release succinate in response to metabolic stress and whether this differs among endothelial subpopulations are open questions. In fact, how endothelial cells switch their metabolism in response to stress, and how this reflects on their functionality and behavior, is an understudied area to date.

While targeting HIF1α might be an attractive target to modulate angiogenesis in diseases like cancer, caution must be taken to not target other HIF isoforms such as HIF2α, which might be part of the defense machinery against disease progression. Indeed, the two isoforms can induce different or even opposite effects. Additionally, because of the complexity of SUCNR1 signaling, which also appears to be context- and cell-type-specific, more efforts are needed to delineate the architecture and the signaling machinery of the succinate receptor in endothelial cells. This approach will enable structure-based drug discovery and might increase the translational potential of current research.
